# A Comparative Study of Aluminium and Titanium Warm Sprayed Coatings on AZ91E Magnesium Alloy

**DOI:** 10.3390/ma15062005

**Published:** 2022-03-08

**Authors:** Rafał Maksymilian Molak, Bartosz Morończyk, Ewa Ura-Bińczyk, Zbigniew Pakieła, Wojciech Żórawski, Krzysztof Jan Kurzydłowski, Seiji Kuroda

**Affiliations:** 1 Faculty of Materials Science and Engineering, Warsaw University of Technology, Wołoska 141, 02-507 Warsaw, Poland; bartosz.moronczyk.dokt@pw.edu.pl (B.M.); ewa.ura@pw.edu.pl (E.U.-B.); zbigniew.pakiela@pw.edu.pl (Z.P.); 2 Faculty of Mechanical Engineering, Bialystok University of Technology, Wiejska 45c, 15-351 Bialystok, Poland; k.kurzydlowski@pb.edu.pl; 3 Faculty of Mechatronics and Mechanical Engineering, Kielce University of Technology, Tysiąclecia Państwa Polskiego 7, 25-314 Kielce, Poland; ktrwz@tu.kielce.pl; 4 Research Center for Structure Materials, National Institute for Materials Science, Tsukuba 305-0047, Japan; kuroda.seiji@nims.go.jp

**Keywords:** warm spraying, thermal spraying, aluminium coatings, titanium coatings, corrosion resistance, magnesium alloys

## Abstract

Aluminium (Al) and titanium (Ti) coatings were applied on AZ91E magnesium alloy using a low-pressure warm spray (WS) method. The deposition was completed using three different nitrogen flow rates (NFR) for both coatings. NFR effects on coating microstructure and other physical properties were systematically studied. Microstructural characterization was performed using scanning electron microscopy (SEM), and the porosity was estimated using two methods—image analysis and X-ray microtomography. The coating adhesion strength, wear resistance, and hardness were examined. The protective properties of the coatings were verified via a salt spray test. Decreasing NFR during coating deposition produced more dense and compact coatings. However, these conditions increased the oxidation of the powder. Al coatings showed lower hardness and wear resistance than Ti coatings, although they are more suitable for corrosion protection due to their low porosity and high compactness.

## 1. Introduction

The low density of magnesium (Mg) alloys makes them an attractive alternative to aluminium (Al) alloys for various structural applications, especially in aerospace. Magnesium alloys offer reduced structure mass, making aeroplanes and other vehicles more economical and thus reducing the environmental pollution. However, the widespread use of magnesium alloys is limited due to their low corrosion resistance and susceptibility to abrasive wear [1]. Magnesium has a low standard potential (E° = −2.363 V/SHE (standard hydrogen electrode), making it highly susceptible to galvanic corrosion. Under atmospheric conditions, the surface of magnesium is covered with magnesium oxide, and the presence of moisture will form magnesium hydroxide. These compounds are stable in a dry and airy environment. Still, they are dissolved in the presence of acids, ions (e.g., chloride, bromine, sulphate, and chlorate), or in water containing acidic gases (e.g., CO_2_). Therefore, much scientific research is currently being conducted to develop an effective and, at the same time, an environmentally friendly method of their protection against corrosion.

The surface anti-corrosion processes, commonly used in industry, including chromate conversion coatings, phosphate, and anodic paint coatings (i.e., conversion coatings), provide limited corrosion protection [2,3]. Chromate conversion coatings [4] are among the most commonly used processes in the aircraft industry, where some magnesium alloys are used (i.e., AM80, AZ91, and AZ31). Coatings produced in this process are diffusively bound to the substrate. However, while effective, these coatings require constant renovation and replacement. Additionally, chromate conversion coatings are formed using chromium (VI) compounds, a known carcinogen and harmful compound to the natural environment [5,6] that will soon be forbidden by EU law [7]. Although magnesium alloys can be anodized similarly to Al alloys, the as-formed coatings are irregularly porous. However, these coatings can be sealed or top-coated for additional corrosion protection [8].

Thermally sprayed coatings are an alternative solution for efficiently protecting magnesium alloys. However, strong corrosion performance is not the only requirement of applied coatings, as reasonable wear, adhesion to the substrate and a homogenous thickness is needed. Several thermal spray techniques have been explored using Ti and Al on various substrates [9,10,11,12], including magnesium alloys [13,14,15,16]. For said application, it should be noted that the produced coatings should be characterized by low porosity and low oxides content to be an effective barrier to environmental impact.

In high-velocity oxy-fuel (HVOF) spraying, the temperature range of deposited particles is approximately 1500–2500 K, which is much too high for Al and titanium (Ti) powders due to the possible oxidation degradation of the deposited material. High processing temperature is especially critical in the case of Ti because a hard and brittle continuous layer of the oxygen-stabilized phase called α-case can be formed. An increasing oxygen level strengthens the α-case and changes the deformation behaviour of α titanium from a wavy to a planar slip mode. Therefore, the hard, less ductile α-case can result in the formation of surface cracks under tension loading, which reduces plasticity [17]. Although the HVOF is mentioned as competitive to WS due to its high process temperature, it is not used to spray pure Al and Ti metals in practice. It can be used only for spraying metals with much higher melting points, such as intermetallic powders based on TiAl [18,19]. The high HVOF spray temperature was the main reason for developing the WS system to control the spray temperature over a wide range.

One of the most explored thermal spray techniques nowadays for forming both Ti [20,21] and Al [22,23] is cold spray (CS) [24,25]. The significant advantage of this method is that it can form coatings using propellant gas at low temperatures (300–600 K) and avoid oxidation of the coating while operating under atmospheric conditions. One of the limitations of the process is the difficulty in producing dense high-strength metal and alloy coatings because of the high critical velocity required to form bonds between deposited particles.

Another technique with solid potential for Ti and Al coating formation is warm spray (WS), an HVOF system modification developed and commercialized by the National Institute for Materials Science (Tsukuba, Japan) [26,27]. This method provides the advantage of process temperature control in the range of approximately 700–1900 K. In WS, the temperature of supersonic gas flow generated by fuel and oxygen combustion is controlled by nitrogen injection. The process takes place in the mixing chamber located between the combustion chamber and the powder feed ports. Metallic powder materials can be deposited in a thermally softened state at high impact velocity. It allows the forming of dense coatings with limited oxidation (in contrast to HVOF) and very low porosity. Since magnesium has a low electrochemical potential compared with most metals, it typically serves as the anode when galvanically coupled with other metals. Therefore, metal coatings on a magnesium substrate must be free from pores to prevent contact with the surrounding environment. Otherwise, Mg corrosion will accelerate at small-scale defect sites due to pitting corrosion [28].

Coatings deposited by WS are a potential way to increase magnesium alloy corrosion performance. Therefore, corrosion resistance in salt spray conditions related to the microstructure, adhesion, and wear properties of Ti and Al coatings deposited by WS were investigated. The study’s primary aim was to identify more suitable ways to protect commercial magnesium alloy AZ91E against harmful environmental effects.

Therefore, adequate corrosion protection of magnesium alloys is essential, and the WS method provides new opportunities in this area. Moreover, the corrosion protection properties of salt spray conditions of Al- and Ti-coated Mg alloys using the WS method have not been investigated so far and represent a novelty. Additionally, the correlation of these properties with microstructure, adhesion and wear resistance of such coatings will allow their application on alloys in industry.

## 2. Experimental

### 2.1. Materials

**Figure 1:**
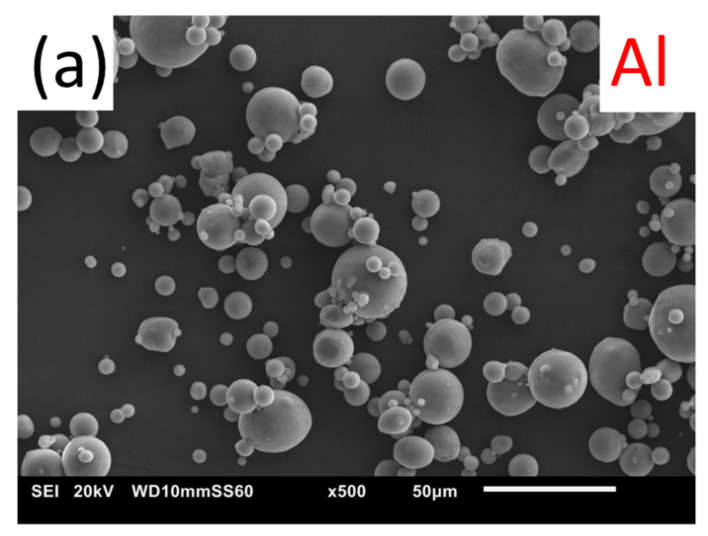

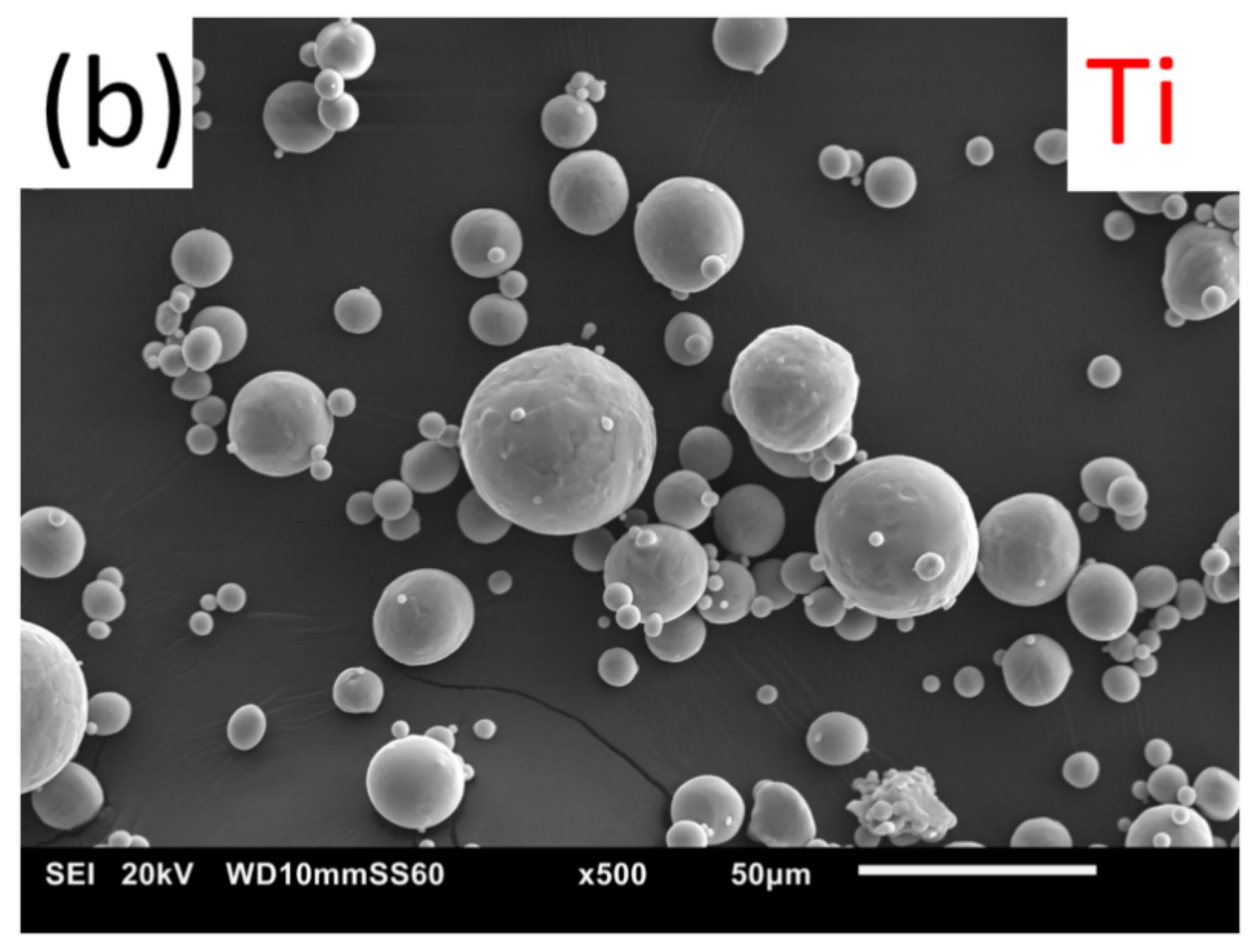
SEM images of Al (**a**) and Ti (**b**) feedstock powder used to produce WS coatings.

**Figure 2:**
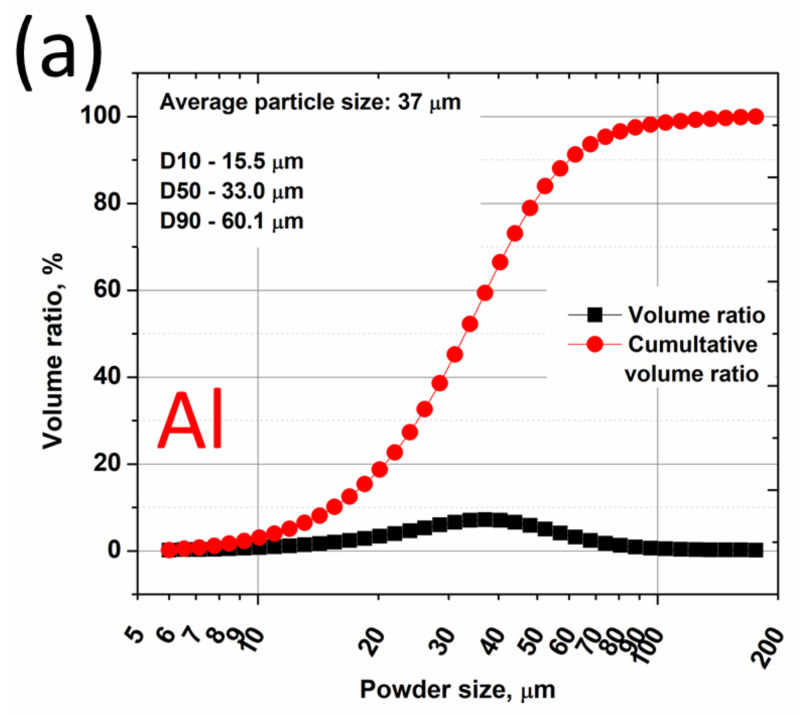

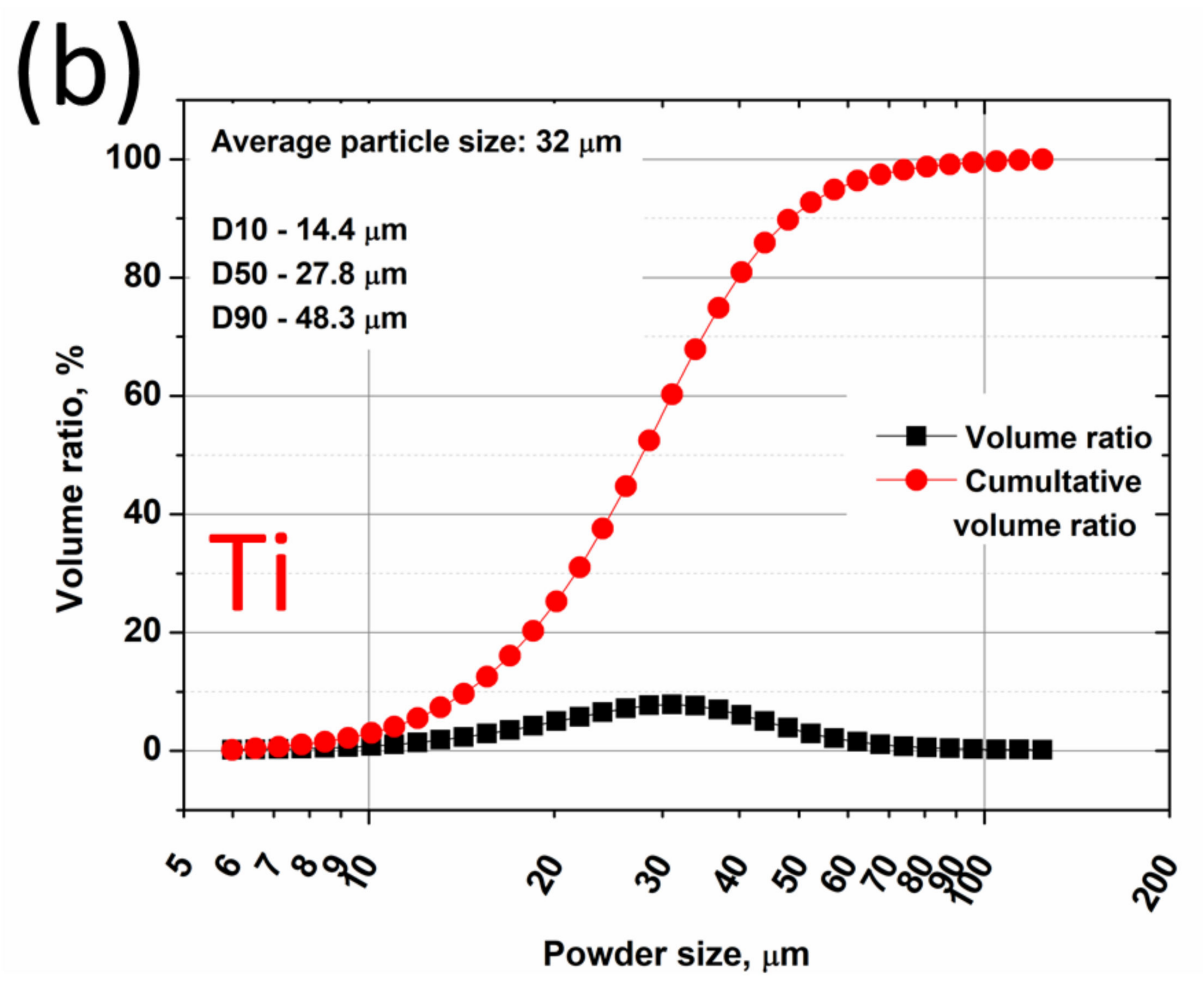
Al (**a**) and Ti (**b**) feedstock powder particle size distribution.

An as-cast AZ91E magnesium alloy, supplied by Pratt and Whitney Rzeszów (Rzeszów, Poland), was used as a substrate. The material was provided in 100 × 50 × 10 mm sand-casted billets. Before coatings deposition, the substrate was grit blasted with alumina particles and cleaned with acetone in an ultrasonic bath US-4R (AS ONE, Osaka, Japan). White fused alumina grit Fujirundum WA36 (Fuji Manufacturing, Tokyo, Japan) with the median diameter in the range of 425 to 600 μm as an abrasive was used. Two commercial powders (TLS Technik GmbH & Co., Bitterfeld-Wolfen, Germany), Ti (grade 2, 99.8 wt% Ti, −45 μm) and Al (99.7 wt% Al, 45 μm), were used for coatings fabrication. Particle size distribution was verified using scanning electron microscopy (SU 8000 Hitachi, Tokyo, Japan) and laser diffraction according to ISO 13320 test specification. A Microtrac S3500 (Retsch GmbH, Haan, Germany) measured particle size and size distribution. Images of the powders are presented in Figure 1, and the measured particle size distribution is shown in Figure 2.

Both powders used in this study were manufactured via gas atomization, which determined the near-spherical morphology of the powder particles (Figure 1). The parameters of D10, D50, and D90 were for Al and Ti powders 15.5, 33, 60.1 μm and 14.4, 27.8, 48.3 μm, respectively, indicating that the size distribution of the powders was similar. The average diameter of both powders was 37 and 32 μm for Al and Ti adequately.

### 2.2. Warm Spraying Process

**Figure 3:**
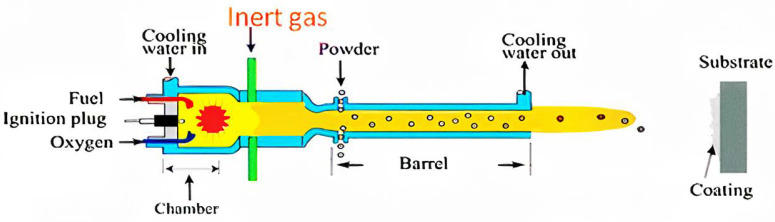
Schematic of the WS gun [29]. Reproduced with permission from Kuroda, S. et al., Journal of Thermal Spray Technology; published by Springer Nature, 2011.

A schematic of the WS process is illustrated in Figure 3. The process parameters are listed in Table 1. The coatings were deposited under different NFR and subsequently named Al_1.0, Al_1.5, Al_2.0 for Al coatings and Ti_0.75, Ti_1.00 Ti_1.25 for Ti coatings, where the numbers represent the NFR during deposition. The mixture’s ratio of kerosene to oxygen was fixed at the stoichiometric level for complete combustion. For increasing NFR, fuel and oxygen flow control was necessary to maintain constant combustion pressure at 1 MPa. WS’s other parameters, such as spray distance, barrel length, and powder feed rate, were fixed in this experiment.

### 2.3. Microstructural Characterization

Cross-sections of the deposited Ti and Al coatings were polished with the SiC paper (Lam Plan, Gaillard, France), followed by 3 μm and 1 μm water-free diamond suspensions (Lam Plan, Gaillard, France) for SEM observation. Imaging and chemical analysis were performed using a SU8000 (Hitachi, Tokyo, Japan) SEM equipped with an energy dispersive spectrometer (EDS) (Thermo Electron Corporation, Waltham, MA, USA). Coating porosity and thickness were analyzed using quantitative image analysis software ImageJ developed at the National Institutes of Health and the Laboratory for Optical and Computational Instrumentation (1.48, University of Wisconsin, Loci, WI, USA). Ten representative SEM images for each WS coating were analyzed to ensure statistical robustness. Additionally, the porosity was measured using X-ray microtomography (Xradia XCT-400, Zeiss, Pleasanton, CA, USA) to determine mechanical polishing effects on the revealed porosity level. In addition, 2 mm × 2 mm specimens taken from a cross-section at half thickness of the coating were examined using 80 kV accelerating voltage and 125 μA current intensity. A phase-contrast enhancer detector was also used, and the samples of 0.9 mm × 0.9 mm were analyzed. Xradia software (XMControler 8.1.6599, Zeiss, Pleasanton, CA, USA) was used to reconstruct final images from slices. Subsequently, all images were processed using Avizo Fire (Thermo Fischer Scientific, Hillsboro, OR, USA), which allowed the porosity volume determination. The resolution of the method was about 2.5 μm.

It should be remembered that standard metallography may be suitably provided smearing and inadvertent material removal can be avoided during polishing. Smearing is particularly common in coatings formed from ductile materials, such as pure Al and Ti. Therefore, polishing quality is a primary source of potential error for subsequent porosity measurements via quantitative image analysis. Compared standard metallography procedure to 3D computational methods provide a more accurate porosity assessment due to the direct observation within the material.

### 2.4. Roughness

The coating surface roughness was evaluated using a Surftest SJ-210 series contact profilometer (Mitutoyo Corporation, Sakado, Japan). The measuring speed and force were 0.5 mm/s and 4 mN, respectively. The tests consisted of measuring three separate lengths of 10 mm each. The standard roughness parameters, R_a_ and R_z_, were evaluated.

### 2.5. Microhardness Test

Microhardness measurements (HV_0.5_) were performed on mirror-finished coating cross-sections using a semi-automatic microhardness tester (MMT-X7B, Matsuzawa, Japan) equipped with a Vickers indenter (Zwick GmbH & Co, ZHU2.5, Ulm, Germany). At least 10 indentations were completed with 5 N loading force for each specimen representing a specific WS condition. The loading velocity was 0.5 N/s, and the sample was held under the maximum load for 15 s.

### 2.6. Adhesion Test

Adhesion measurements were completed under the guidelines of ASTM C633-13 “Standard Test Method for Adhesion or Cohesion Strength of Thermal Spray Coatings”. A pull-off test where tension is normal to the surface plane of the coating, allowing for determination of the adhesive or cohesive coating strength, was performed. However, the standard recommended specimen diameter of 1” was reduced to 7 mm to conserve testing material. Cylindrical specimens were cut from plates deposited with WS using wire electric discharge machining (AgieCharmilles Robofil 290P, GF Machining SolutionsSękocin, Nowy, Poland).

Coatings were bonded to the counter-specimen using epoxy adhesive DP490 (3 M Company, Saint Paul, MN, USA). The Al6061 alloy counter-specimen surface was roughed using P600 grit SiC paper (Lam Plan, Gaillard, France), and both parts were degreased before the bonding process using acetone. A few 200 μm diameter glass spheres were added to the adhesive volume during bonding to ensure optimal bonding thickness after applying pressure. The bonded specimen was then clamped in the proper pneumatic grip of the testing machine. The turnbuckle was mounted to ensure the axiality of the testing stand between the upper testing machine clamping point and the testing specimen. The lower part of the specimen was clamped using pneumatic grips of the testing stand. The bonding procedure consisted of a 24-h room temperature cure, followed by a 1 h 80 °C treatment. A Zwick/Roell Z005 (Zwick GmbH & Co, Ulm, Germany) universal testing machine with a ±5 kN load cell and crosshead displacement at 2.5 mm/min was used for testing.

### 2.7. Wear Test

The wear resistance of the deposited coatings was evaluated by a modified pin-on-disc method under dry conditions using a tribotester (labelled T-17) (Łukasiewicz Research Network, The Institute for Sustainable Technologies, Radom, Poland). Tests were designed according to the ASTM G-99 “Standard Test Method for Wear Testing with a Pin-on-Disk Apparatus”. However, in our case, a reciprocating motion was applied due to the design of the testing stand. A sliding pair consisted of a stationary pin that applied a force of 2 N onto the plate and moved back and forth. A 1 Hz test frequency and 8 mm sliding path amplitude were applied. The total test time was approximately 1 h or 115 m of sliding distance. The 9 mm diameter counterpart pins were made using heat-treated 1.2063 steel with 700 HV_10_ hardness. Both surfaces used in the wear test were cleaned with acetone and weighed before and after testing. No additional machining was carried out before the wear test. Weight measurements were conducted using a balance to determine the volume of material used. After wear tests, the surface and sample cross-section images were also documented using a Tagarno FHD Prestige light microscope (Tagarno, Horsens, Denmark) and 3500 SEM (Hitachi, Tokyo, Japan).

### 2.8. Salt Spray Test

For the neutral salt spray test, 1.5 × 1.5 cm sealed specimens were exposed to spray fog for 96 h according to the ASTM B117 “Standard Practice for Operating Salt Spray (Fog) Apparatus”. The epoxy-based polymer labelled Rockhard 576-450-002-R1 (Indestructible Paint Limited, Birmingham, UK) was used as a sealer. Both sides of the specimen were covered to ensure a 1 cm^2^ exposed area. The sealing process proceeded by applying three layers of epoxy-based resin. The sealing procedure was based upon the standard method industrially used. The first layer was applied by dipping the samples in a 4:1 mixture of resin and dedicated thinner for 5 min to ensure infiltration of the coatings’ pores. Then, samples were air-dried for 10 min and then placed in a 185 °C oven for 15 min. Two additional resin layers were applied in the same way, except that the oven time was extended up to 60 min. A S1000 salt spray chamber (Weiss Technik, Reiskirchen, Germany) was utilized for testing. A 5.0 wt% NaCl salt spray solution was used, and the chamber temperature was maintained between 35–37 °C. Two samples for each coating were tested.

## 3. Results and Discussion

### 3.1. Microstructure Characterization

**Figure 4:**
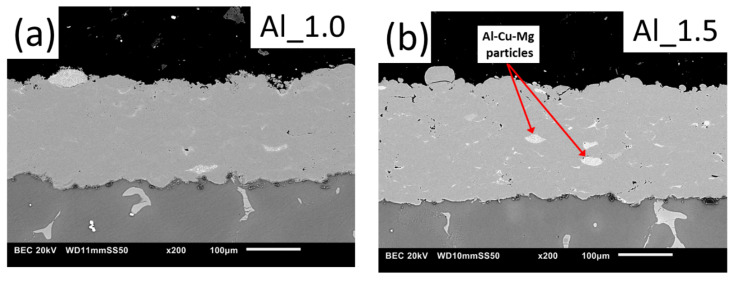

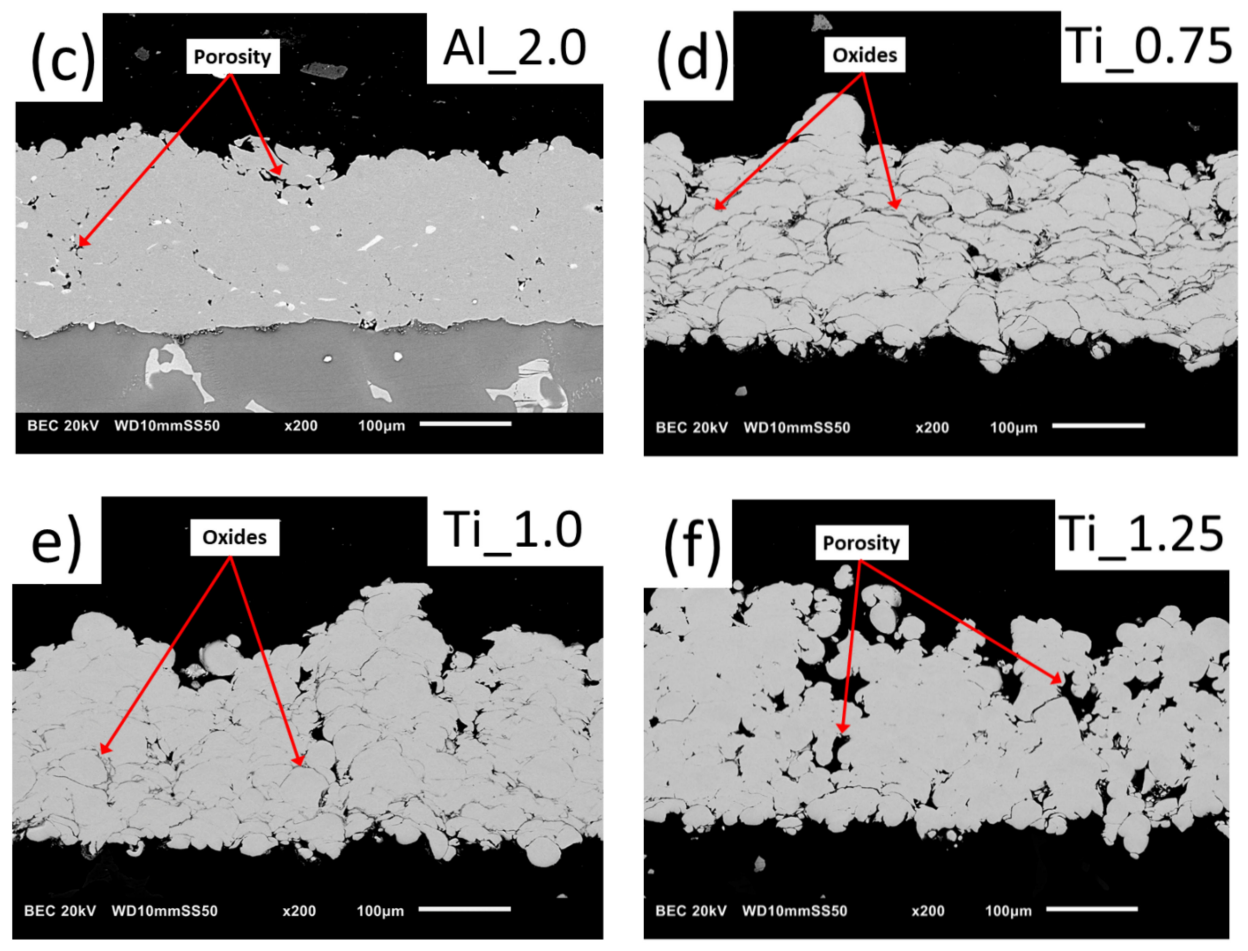
SEM images of cross-sections of WS coatings: (**a**) Al_1.0, (**b**) Al_1.5, (**c**) Al_2.0, (**d**) Ti_0.75, (**e**) Ti_1.0, and (**f**) Ti_1.25.

The cross-sectional observations of Al and Ti coatings deposited using three NFR during WS are presented in Figure 4. The thickness of Al and Ti coatings was 203 ± 10 µm and 222 ± 9 µm adequately, which confirms the assumed spraying parameter accuracy. In general, the cross-sections observed using backscattered electron (BSE) detection contained three grayscale contrast ranges. Light grey regions represent the inner region of deposited particles. Dark regions are correlated with oxides, often formed at the particle surface. Black areas indicate material discontinuities such as pores, microcracks, and unbonded surfaces of the particles.

**Figure 5:**
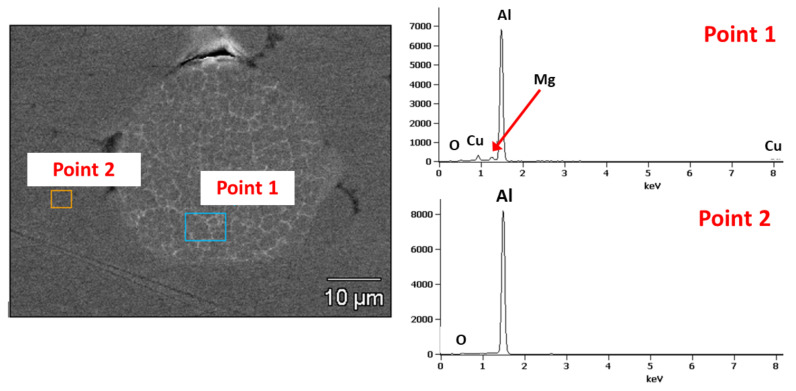
EDS analysis of contamination particles in Al coatings.

Regardless of the parameters used, the Al coatings are denser and more uniform than Ti coatings. The Al coatings show a uniform, compact structure without distinct boundaries and oxide layers between deformed particles. A few bright particles are visible inside the deposited coatings due to the delivered powder. EDS point analysis (Figure 5, Point 1) showed Al-based particles (more than 90 wt%) with about 5 wt% Cu and 1 wt% Mg. The said composition indicates that these may be Al alloy of the 2000 series. The powder contamination is probably from the powder manufacturing process, as indicated by similar observations made for the powder before the spraying process.

Both coatings were affected by the NFR. In general, coatings prepared with the lowest NFR, which resulted in higher temperature, exhibited a denser microstructure (Figure 4a,d), but oxides at particle boundaries were observed, especially in the case of Ti coatings deposited at Ti_0.75 and Ti_1.0 (Figure 4d,e, respectively). The difference in coating porosity was challenging to visually observe in Al coatings and was much more noticeable in Ti coatings. This effect is due to the lower yield stress of Al compared with Ti, which determines plastic deformability and the ability to form a dense coating. Due to the high plasticity of Al, negligible porosity is obtained regardless of the spraying conditions. Ti coatings also showed differences in microstructure for variable NFR. Increasing NFR, which decreases deposition temperature, resulted in more uniform coatings in terms of phase. However, the porosity clearly increased, as seen in the expanding black area in the BSE SEM cross-sections. On the other hand, the Ti coating surface applied at high NFR decreased oxidation due to the reduced process temperature (Figure 4f). It should also be noted that no oxidation was observed in Al coatings, even for the highest process temperature (Figure 4a (Al_1.0)). The above observations, especially for Ti, are consistent with our previously published results [30,31,32]. The dense and uniform Al coatings are more suitable for better corrosion resistance, unlike the porous and oxidized Ti depositions.

**Figure 6:**
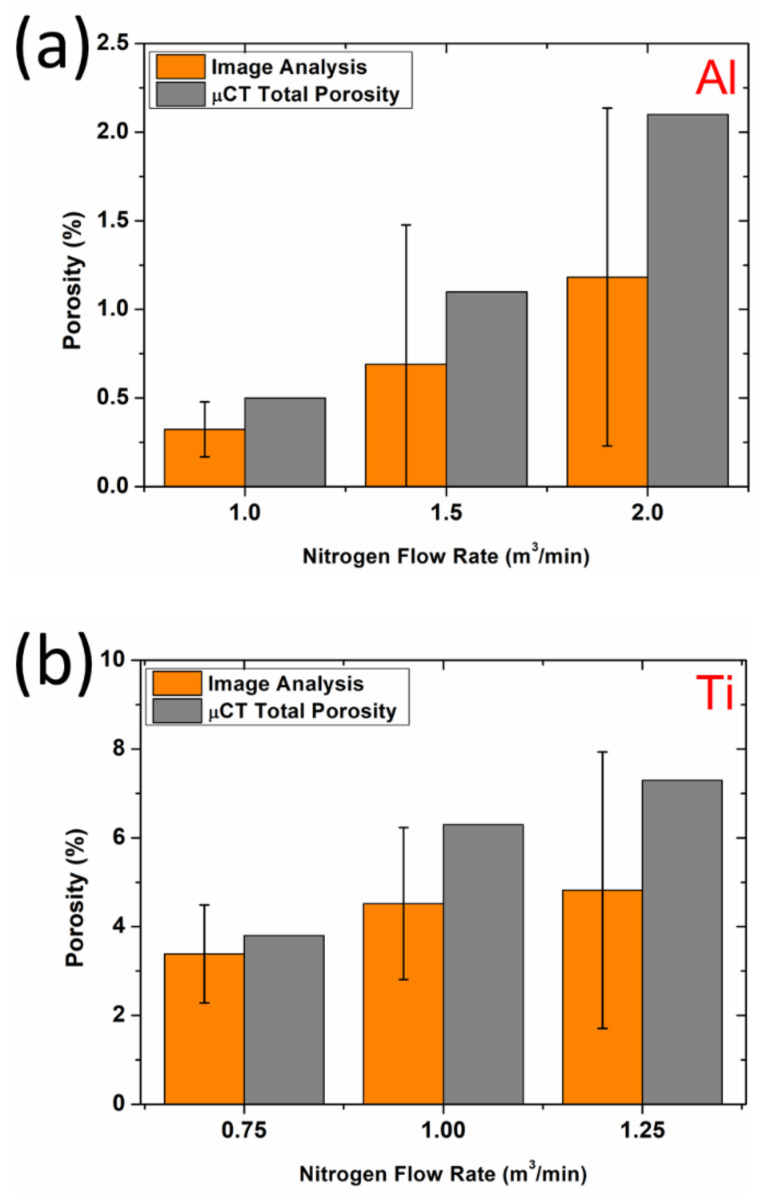
Porosity of Al (**a**) and Ti (**b**) coatings.

Porosity analysis of the obtained coatings was performed to confirm the above examinations, and the results are shown in Figure 6a,b for Al and Ti, respectively.

The porosity increased with increasing NFR for all analyzed coating materials regardless of the analysis method. The determined values confirmed that the Ti coatings were significantly more porous in the full range of applied parameters. The average total porosity measured by µCT was higher than for image analysis for all coatings. All values calculated by image analysis were determined using 10 cross-sections.

The surface preparation method, especially the applied force during grinding and polishing, plays a meaningful role in the visible, apparent porosity on the coating cross-sections [33,34]. For ductile materials (i.e., Al and Ti), coatings can deform during metallographic preparation, reducing the amount of detectable porosity during image analysis [35]. Surface pores are closed due to severe plastic deformation. Meanwhile, µCT studies performed in the material volume render these kinds of preparation effects negligible. As such, µCT analysis was used to measure the total porosity and pore distribution throughout the entire sample, not only the surface, as demonstrated in our previous studies regarding Al [36] and Ti [37] coatings. However, the µCT is limited by resolution and studied area size, both of which are lower than image analysis. Rolland et al. [38] mentioned a similar trade-off when comparing these two methods. Our 2D and 3D results overlap within the error bar for 5 of 6 results.

### 3.2. Roughness

**Figure 7:**
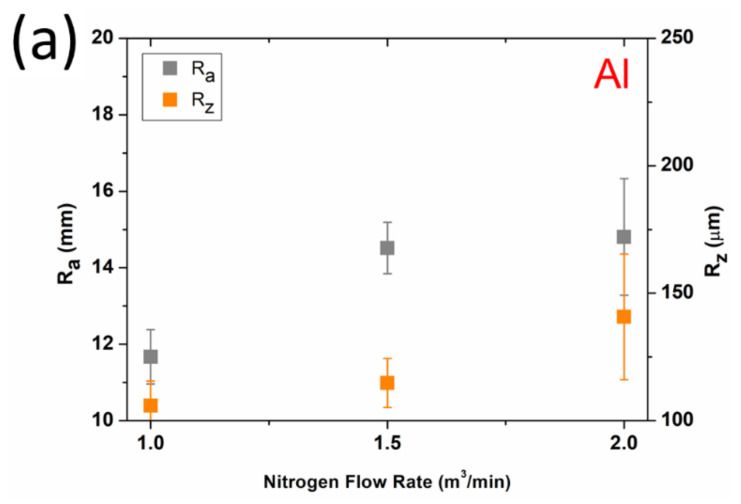

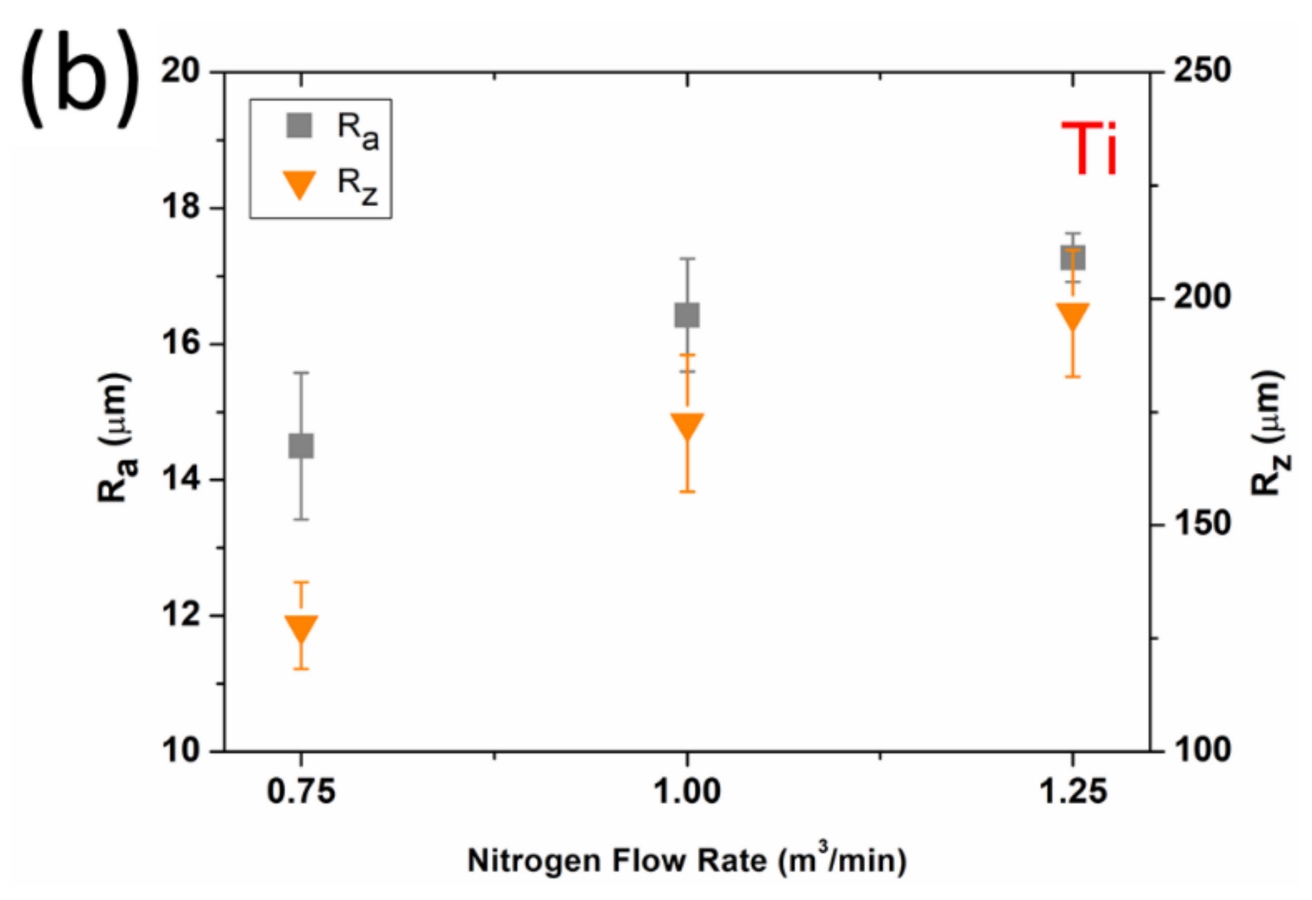
Surface roughness of Al (**a**) and Ti (**b**) coatings.

The surface roughness measurement results are presented in Figure 7a,b for Al and Ti. Ti coatings generally showed higher surface roughness than Al coatings, independent of spray parameters, due to the higher yield stress and subsequent lower capacity for plastic deformation of Ti particles. Both materials showed a general decrease in coating roughness with decreasing NFR due to increased process temperature. Due to thermal softening effects, deposited particles became increasingly flattened with increasing WS temperature. This observation is consistent with our previous studies [30], where the geometry (splats flattening ratio) of single deposited particles made of Ti6Al4V were analyzed as a function of gas velocity and temperature. However, we note that temperature is the primary determinant of particle plastic deformation. Numerical simulations previously performed [27,39] for 30 μm Ti particles sprayed with low-pressure WS showed that NFR mainly affects particle temperature, ranging from 750 to 1500 K. Interestingly, the particle velocity profiles were unaffected by NFR. Thus, increasing the flame stream density by adding nitrogen compensates for the decreased gas velocity resulting from reduced propellant gas temperature.

**Figure 8:**
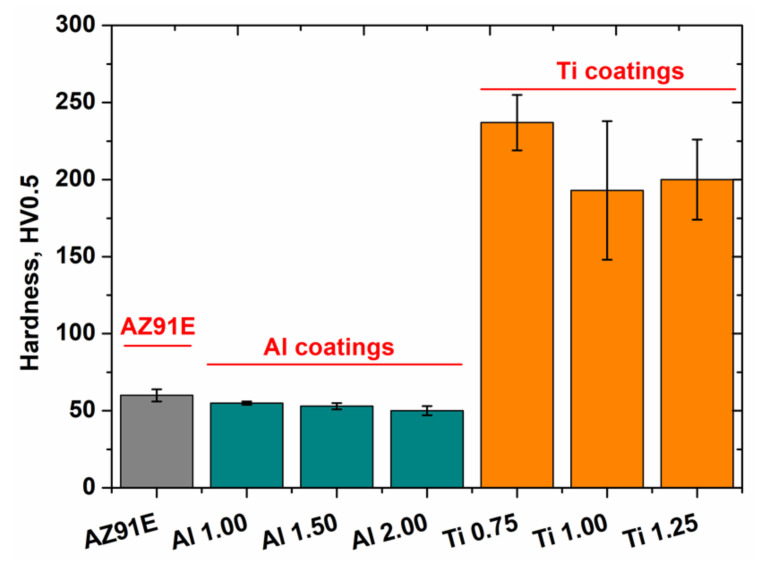
Hardness of Al, Ti coatings and AZ91E substrate plate.

Vickers hardness values (HV0.5) of the WS coatings are shown in Figure 8, along with the plate substrate (AZ91E) microhardness measurements. The results show decreasing microhardness as a function of increasing NFR. This effect is visible for both Al and Ti. Samples formed at the highest temperature (using 1.0 m^3^/min NFR for Al and 0.75 m^3^/min for Ti) exhibited the highest microhardness. For low NFR in WS deposition, spray particles formed at a higher processing temperature and thermally softened compared with other WS conditions, producing more extensive deformation upon impact associated with work hardening. Strain hardening was also observed in cold spraying (CS), a related coating application method [40,41,42]. The microhardness increase was attributed to the strain hardening phenomenon during deposition [43,44].

Oxidation was another factor behind the observed increased microhardness. For high particle temperatures, particles are significantly oxidized to produce a thick outer oxide layer, especially in the case of Ti coatings formed at 0.75 NFR (Figure 4d). The high affinity of Ti towards oxidation combined with the high solid solubility of oxygen in Ti (14.5 wt%) resulted in forming an oxygen-rich α-case layer [45]. Increasing oxygen concentration strengthens the α-case, making this phase harder than the primary phase.

The third contribution to the effect discussed above is that particles cannot deform enough upon impact at low temperature, resulting in a porous microstructure. Additionally, the weak bonding strength between deposited particles contributes to reduced microhardness. Since the temperature was a primary factor affected by NFR changes, decreased hardness can be attributed to a weakened mechanical bond between the coating particles.

### 3.3. Salt Spray Test

**Figure 9:**
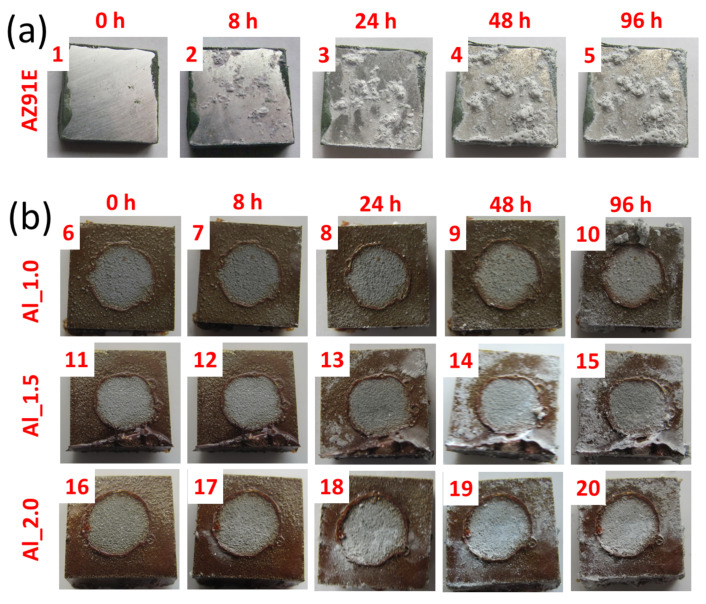

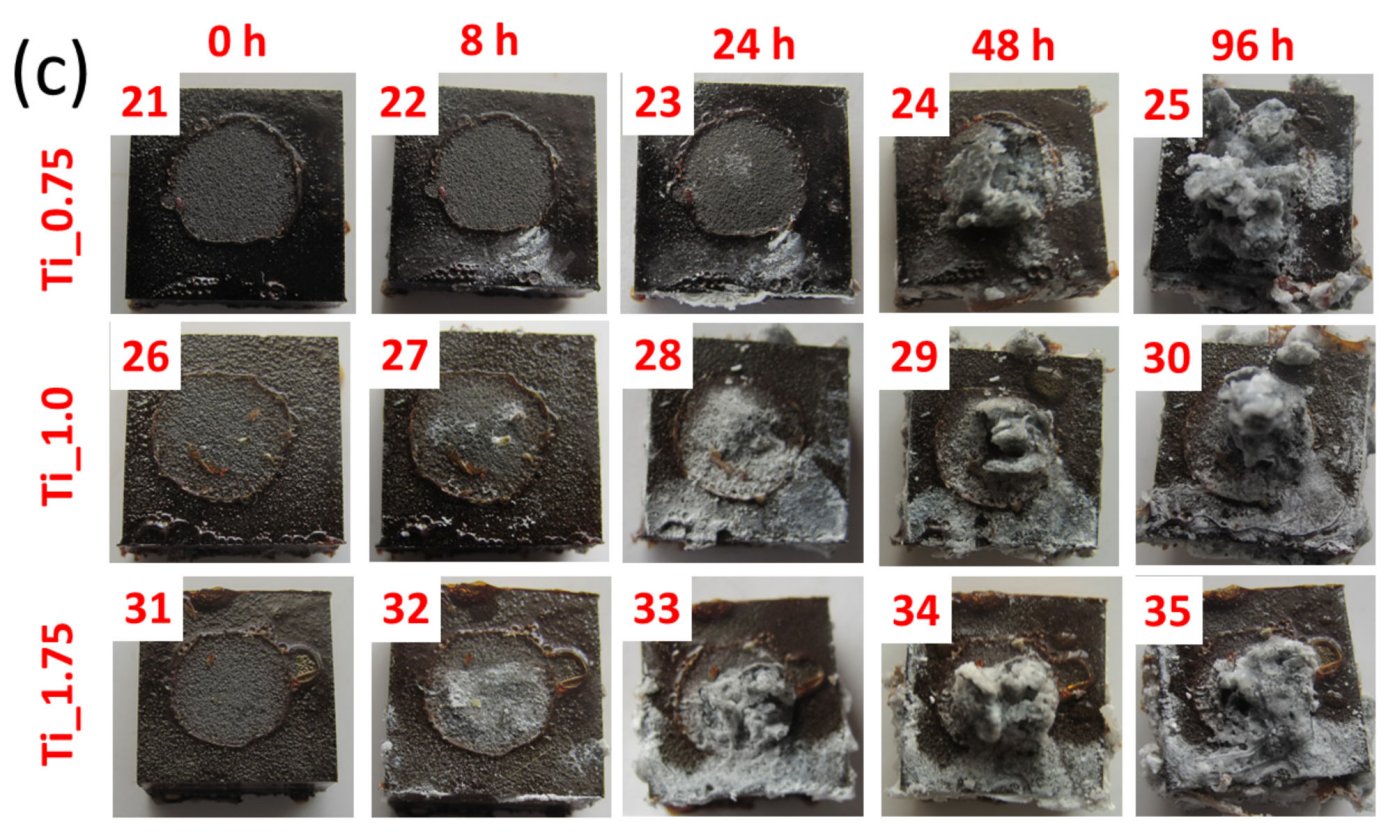
Surface observations at 0, 8, 24, 48, and 96 h of salt spray corrosion test for AZ91E substrate plate (**a**), WS Al (**b**) and Ti (**c**) coatings.

The macro morphologies of the AZ91E alloy substrate and Al and Ti coatings before and after salt spray testing are shown in Figure 9. The sides and edges of the coated samples were protected with polymer sealing to prevent corrosion of the bare substrate. Before corrosion testing, the raw substrate and WS coating substrates were relatively uniform and homogenous. For AZ91E, corrosion products first appeared after 8 h of salt spray tests (Figure 9a, no. 2) and then increased with increasing exposure time (Figure 9a, Nos. 3–5). 

All Al coatings provided corrosion protection throughout the 96 h test period. The Al coating formed at 1.5 NFR showed minor traces of corrosion, but the corrosion process started at the sample edge (Figure 9b, no. 14), the most difficult area to protect with resin.

In the case of Ti, coatings with significant porosity (Ti_1.25 and Ti_1.0) only protected the substrate for 8 h (Figure 9c, no 27 and 32, respectively). The high porosity allowed for salt penetration, and the substrate showed signs of corrosion after only a few hours. The coating formed at the highest temperature (Ti_0.75) and characterized by the lowest porosity (Figure 6b) lasted about 24 h (Figure 9c, no. 23). After a specified critical time, a rapid increase in the pitting corrosion rate is observed in all Ti coatings due to the loss of coating continuity. Comparing the volume of corrosion products on the AZ91E sample (Figure 9a, no. 5) and Ti coatings after 96 h, the cathodic Ti coatings accelerated corrosion of the anodic substrate, as a much larger corrosion product volume was observed on Ti coatings (Figure 9c, Nos. 25, 30, and 35).

Due to the limited availability of the WS systems, few investigations study corrosion protection of coatings produced by this method. Among them are those published by the authors and relating to Al [36] and Ti [37] coatings produced by WS. A relationship between porosity, oxygen content, and NFR was revealed for Ti coatings. Corrosion testing using a 3.5 wt% NaCl solution of as-sprayed coatings revealed poor corrosion resistance, leading to rapid substrate degradation due to the significant porosity. Only post-treatment with an epoxy-based polymer substantially improved the corrosion performance of Ti WS coatings. Similar observations were made for Al coatings, but due to the much lower porosity, the effect of spray parameters on corrosion properties was reduced. Moreover, these did not require additional treatments, such as sealing with the epoxy resin, to provide sufficient protection for the magnesium alloy substrate.

Al coatings for the protection of magnesium substrates produced by related methods such as CS have been more widely explored. Galvanic immersion tests and fog spray tests in 5 wt% NaCl solutions were performed to assess corrosion protection effectiveness. Tao et al. used CS to deposit elemental Al coatings on AZ91D magnesium alloy substrates [14]. It was concluded that the corrosion current densities of the coatings were comparable to the elemental Al and a few orders of magnitude lower than that of the bare AZ91D alloy substrate. Wang et al. reported similar results for AZ91E using similar experiments [13]. Diab et al. [46] showed that pure Al coatings provided significant corrosion protection for AZ31B in a 5% NaCl fog environment by improving its corrosion resistance from 90% average weight loss in 33 days for a bare substrate less than 10% average weight loss in 90 days. Although Ti coatings are not as effective as those made of Al, they show adequate corrosion protection under certain conditions. The critical issue is minimizing porosity and ensuring continuity of the applied coating [9,10].

### 3.4. Adhesion

**Figure 10:**
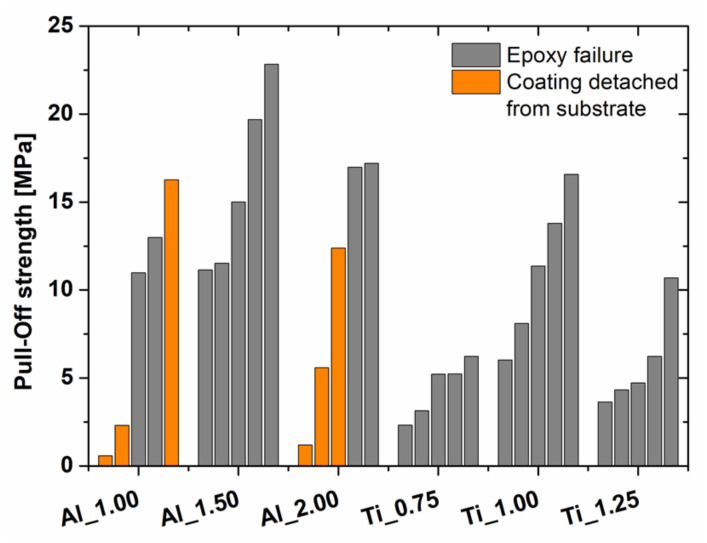
Pull-off strength of Al and Ti coatings.

The pull-off strength of deposited Al and Ti coatings for five independent measurements measured using adhesion tests are presented in Figure 10. In most cases, the trials ended due to epoxy failure at the epoxy–coating interface. Therefore, the exact adhesion strength could not be measured, but the minimal adhesion strength of the studied WS coatings was evaluated. Only six Al coatings were detached from the substrate metal during the test. Coating failure for two Al_1.00 and Al_2.00 samples at very low stress (below 5 MPa) were considered errors during the bonding procedure. All other failures during adhesion testing of the studied Al coatings occurred above 10 MPa stress, indicating relatively good adhesion strength. The two above-mentioned Al coatings, Al_1.00 and Al_2.00, were successfully detached from the substrate at 16 MPa and 12 MPa. In both cases, failure was evident in adhesive mode.

Significantly lower adhesion values were achieved during adhesion testing of Ti coatings. Additionally, none of the tested coatings were successfully detached from the substrate. In most cases, epoxy in samples from Ti_0.75 and Ti_1.25 failed around 5 MPa. The highest pull-off strength was recorded for Ti_1.00, which achieved at least 10 MPa for 3 of the 5 trials.

Due to the complexity of factors affecting the coating adhesion, interpretation of the results was complex and required further in-depth analysis beyond the scope of this paper. Relating the obtained results to other coatings obtained by WS is difficult due to the lack of studies. The only solution is to use knowledge developed for related coatings produced by CS.

The critical condition to achieve minimum adhesion of deposited particles to the substrate is to reach a critical velocity, V_c_. On the other hand, too high impact velocity leads to substrate surface erosion [47] due to excess elastic energy available for particle rebounding. The velocity range limit by the above criteria defines the CS deposition window of a given material [42].

In general, the V_c_ for achieving Al adhesion (500 ÷ 650 m/s [47,48]) is lower than Ti (above 750 m/s [49]). This effect is related to the mechanical properties (strength and yield strength) determined in part by the crystallographic structure and possible slip systems. However, we must also remember that the values mentioned are a function of the applied particle size and the mechanical properties of the substrate material, which may explain the lower adhesion values for Ti coatings compared with Al. Regardless of the spray parameters, coatings applied by WS were produced at similar particle velocities. As explained above, changes in NFR mainly affect the WS process temperature, not the particle velocity.

The results for Al depositions by WS can be compared with CS Al coating adhesion to AZ91E substrate results reported by Tao et al. [14] of about 18 MPa. For Ti coatings, the adhesion strengths in the range of about 15–19 MPa have been reported for steel substrates [50]. Due to the lack of such data, no definite reference could be made to coatings formed on magnesium alloys.

The primary way to increase particle velocity in both WS and CS methods is to increase the process pressure. In our case, this was limited by using a low-pressure WS system with a propellant gas pressure of 1 MPa, resulting in particle velocity of about 850 m/s [51], which is just above the mentioned critical value for Ti. It was the primary reason for the relatively low adhesion value of the coatings to the substrate AZ91E. However, the achieved adhesion values do not rule out the usefulness of the obtained coatings. Furthermore, they are within the ranges reported by other works and can be successfully used where high adhesion values are not critical such as non-structural applications like anti-corrosion coatings for aircraft engine and gearbox covers, air intakes, etc.

### 3.5. Wear Test

**Figure 11:**
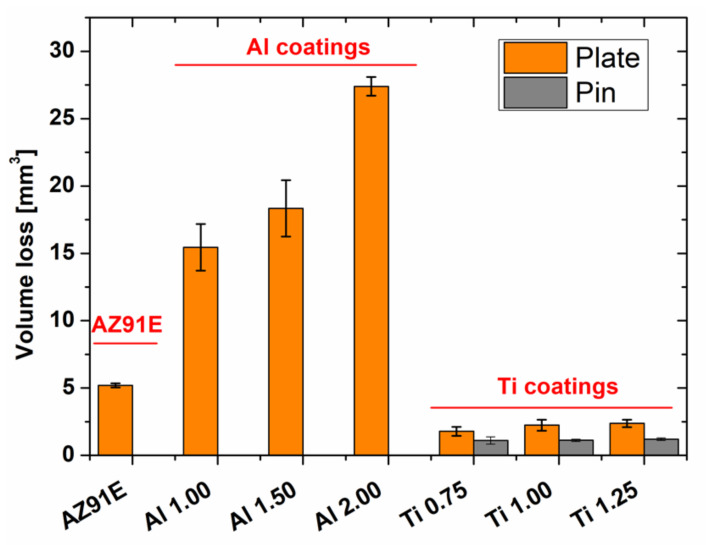
Volume loss of Al and Ti coatings, AZ91E substrate plate and pin material.

Volume loss of the tested coatings and substrate material, measured after pin-on-plate wear tests, is presented in Figure 11. Loss of material for Al coatings is about six times lower than Ti coatings and three times lower than the bare magnesium alloy substrate. Despite their various porosity and hardness, no significant differences in volume loss for WS Ti were found. As the wear mechanism for Ti is mainly adhesive, the wear resistance of Ti cannot be improved by increasing its hardness [52]. In addition, due to the adhesive nature of Ti wear, there was significant wear on the hardened steel pin as the counterpart for Ti deposit trials. Pin volume loss could not be reliably measured for Al coatings and the substrate material due to small values beyond the resolution of the measuring instrument. In Al coatings, there is a clear relationship between deteriorating wear resistance when increasing NFR and decreasing WS process temperature.

**Figure 12:**
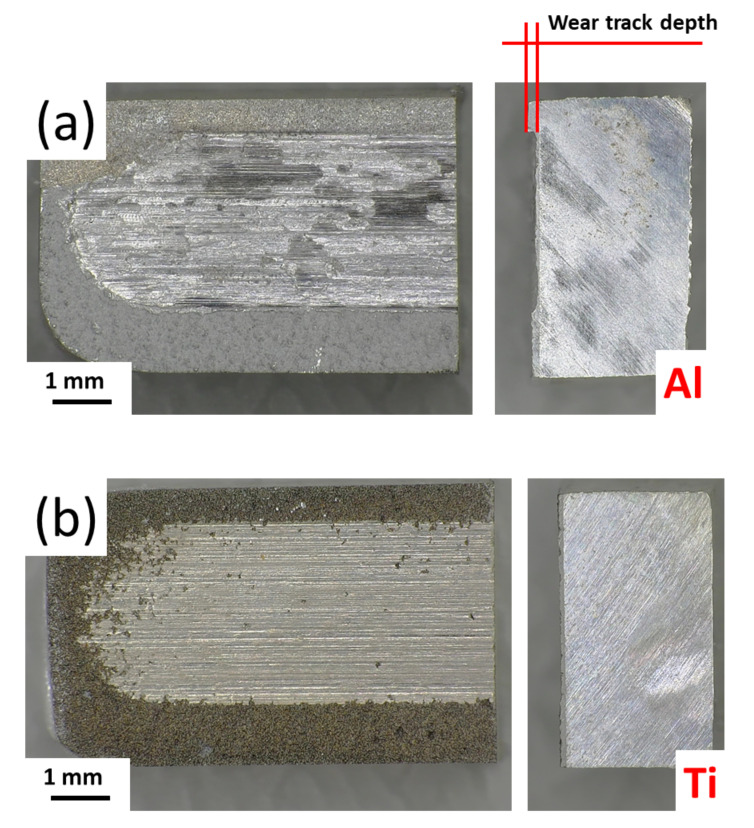
Top and cross-sectional view of Al_1.5 (**a**) and Ti_1.0 (**b**) coatings after pin on plate wear test.

**Figure 13:**
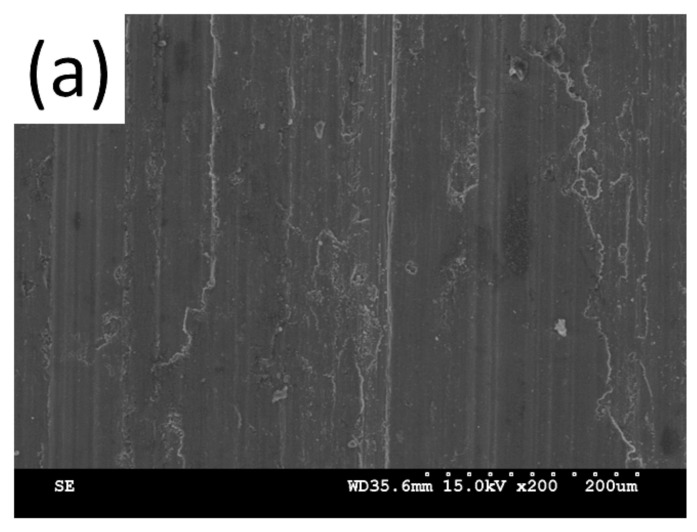

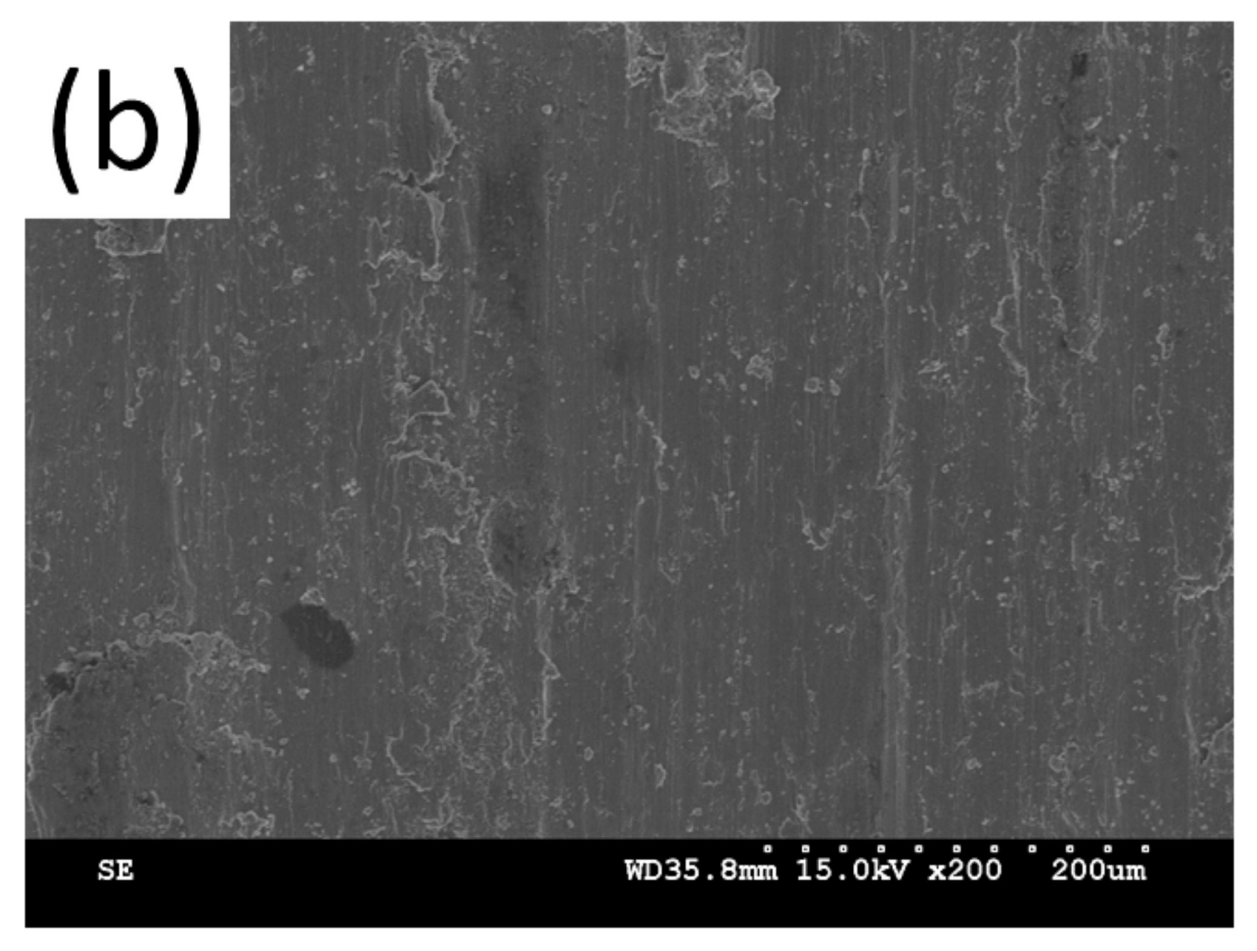
SEM images of wear track for Al (**a**) and Ti (**b**) coatings.

Macro- (Figure 12a,b) and microscopic (Figure 13a,b) observations confirmed the above quantitative analysis of the coating and plate material wear properties (Figure 11). For both the top view and cross-section, significant wear of the Al coating can be seen in contrast to the Ti coating, which showed much better wear resistance regardless of WS spray parameters. In the case of Al coating, the depth of the wear track is visible on the cross-section of the specimen (Figure 12a), which is not the case for the Ti (Figure 12b).

As described during the microstructure analysis, these changes are derivative of two independent phenomena occurring during coating deposition (i.e., strain hardening and thermal softening). The mutual contribution of these phenomena, being a derivative of the WS temperature, which is a function of the NFR parameter, determines the coating microstructure (i.e., porosity, oxidized area) and mechanical properties (e.g., hardness) [30,31,32].

The wear behaviour of Al is complex and varies under different application conditions. Under sliding wear conditions, the wear rate of Al is affected by system factors (i.e., applied load, sliding speed, and type of counterpart material) as well as a material factor (i.e., chemical composition, microstructure, hardness). However, in the case of dry sliding linear wear under metal-to-metal contact, the wear resistance of Al is relatively poor [53].

SEM images of wear tracks for both Al and Ti coatings (Figure 13a,b, respectively) showing a smooth surface with parallel furrows indicate a wear mechanism through plastic flow by accumulated plastic shear flow and was classified as an adhesive wear mechanism [54].

There are no available data for the wear behaviour of WS coatings in the literature. Wear properties of CS coatings are reported rather briefly in the literature. These studies mainly concentrate on the friction of coefficient and sliding wear studies. However, there are reports that Al coatings deposited using CS did not show significantly better wear properties than the Al bulk substrate plate [55]. Due to the limited wear resistance of pure Al, the present activities are focused on ways to improve this property. It can be achieved by using alloying agents, heat-treating the deposited as-sprayed coatings, or producing composite coatings. For instance, Pitchuka et al. [56] reported heat-treated coatings exhibiting higher wear resistance and lower friction coefficient than the as-sprayed coatings. Furthermore, improved wear properties of CS Al-Al_2_O_3_ composite coatings have been reported by Shockley et al. [57] and Spencer et al. [58].

## 4. Conclusions

In this study, a comprehensive comparison of Al and Ti coatings deposited using the WS on AZ91E magnesium alloy has been made. Although both the substrate and metal powders used in the spraying process are sensitive to high temperatures, effective coatings were produced in both cases using the WS. The analysis of the essential functional characteristics of the coatings indicated that Al is characterized by lower porosity and, at the same time, higher uniformity compared with Ti. Lower porosity of Al coatings resulted from lower mechanical properties of Al (e.g., yield strength). On the other hand, higher uniformity of the coatings resulted from lower susceptibility of Al to oxidation under specific deposition conditions. In the case of Ti coatings, apart from higher porosity, the occurrence of single particles oxidation and forming a so-called α-case on the particle surface was observed.

The lower yield strength and higher plastic deformation capacity of Al contributed to the lower roughness of Al coatings. On the other hand, the ability of Ti to strain hardening resulted in much higher microhardness values of Ti coatings. As a natural consequence of the higher porosity, the corrosion protection efficiency of AZ91E alloy by Ti coatings was much lower when compared to Al. Adhesion tests made it possible to determine the minimum adhesion values for both types of coatings; however, difficulties were encountered in this part of the work related to the failure of the adhesive layer at relatively low stresses. On the other hand, the wear resistance tests indicate an advantage of Ti coatings in this respect, which showed much lower wear values compared with Al coatings.

Considering that the primary function of the produced coatings is the corrosion protection of magnesium alloy, Al coatings should be indicated here as being more helpful in this application, despite their lower abrasion resistance. This feature can be improved by increasing the coating thickness.

The following conclusions can be drawn:

1. Increasing nitrogen flow rate (NFR) during WS deposition, which decreases the process temperature, led to increased porosity and surface roughness of studied coatings. NFR had a much more pronounced influence on the Ti coating microstructure than Al.
2. Ti coatings exhibited higher porosity and higher surface roughness than Al coatings due to higher mechanical properties, in particular yield strength, and thus higher requirements for critical velocity V_c_.
3. Despite higher porosity and surface roughness, Ti coatings with higher hardness exhibited much lower volume wear loss in pin on disk wear tests compared to Al coatings and AZ91E substrate material.
4. The performed pull-off test could not determine the adhesion strength of the deposited WS coatings, but the minimal adhesion strength could be evaluated for Al coatings.
5. Ti coating porosity led to poor corrosion resistance in the salt spray test since NaCl solution could penetrate the coating, accelerated galvanic corrosion that occurred at the substrate/coating interface. Al coatings provided strong corrosion protection during 96 h of salt spray test without signs of substrate corrosion.

## Figures and Tables

**Table 1:** WS parameters.

l.p.	Coating	Fuel/Gas Flow [m^3^/min]			Combustion Pressure (Mpa)	Spraying Distance (mm)	Barrel Length (mm)	Powder Flow Rate (g/min)
		Fuel	Oxygen	Nitrogen				
1	Al_1.0	0.34	0.69	1.00	1	200	200	45
2	Al_1.5	0.29	0.59	1.50				
3	Al_2.0	0.27	0.55	2.00				
4	Ti_0.75	0.36	0.73	0.75				
5	Ti_1.0	0.34	0.69	1.00				
6	Ti_1.25	0.32	0.59	1.25				

## Data Availability

Data sharing is not applicable.
